# Mechanobiological Implications of Low–Young’s Modulus TiNbSn Alloy Plates for Fracture Fixation: A Focused Review

**DOI:** 10.3390/medsci14010149

**Published:** 2026-03-19

**Authors:** Yu Mori, Hidetatsu Tanaka, Masayuki Kamimura, Naoko Mori, Toshimi Aizawa

**Affiliations:** 1 Department of Orthopaedic Surgery, Tohoku University Graduate School of Medicine, Sendai 980-8574, Japan; 2 Department of Radiology, Akita University Graduate School of Medicine, Akita 010-8543, Japan

**Keywords:** fracture, TiNbSn alloy, low Young’s modulus, biological fixation, interfragmentary strain

## Abstract

Rigid internal fixation has long been the standard for fracture management; however, excessive construct stiffness can suppress interfragmentary strain, reduce callus formation, and impair secondary fracture healing. Low-elastic-modulus TiNbSn alloys have emerged as a promising alternative, offering mechanical behavior closer to that of cortical bone. This review synthesizes representative preclinical and computational evidence to clarify the mechanobiological rationale for TiNbSn alloy plates in fracture fixation. We summarize key biological requirements for secondary fracture healing, including controlled interfragmentary strain, preservation of vascularity, and effective load sharing, and contrast these with the limitations of conventional high-stiffness fixation plates, such as stress shielding and reduced callus formation. Finite element analyses from previously reported models illustrate qualitative trends toward increased axial displacement, favorable stress distribution, and within a biologically relevant range for endochondral ossification. Consistent findings from animal fracture models further indicate enhanced periosteal and intramedullary callus formation and more physiological healing patterns with TiNbSn plates compared with rigid fixation. Emerging clinical experience with TiNbSn femoral stems provides indirect support for the long-term potential of low-elastic-modulus titanium alloys to mitigate stress shielding; however, such findings should be interpreted only as indirect supportive evidence, as stem implantation and fracture plate fixation involve substantially different mechanical and biological contexts. Collectively, these observations provide preliminary support for the mechanobiological rationale of low-modulus TiNbSn plates and suggest their potential role as biologically informed fixation devices, while highlighting the need for further clinical validation.

## 1. Introduction

Fracture fixation aims to provide sufficient mechanical stability to allow bone healing while preserving the biological environment required for tissue regeneration. Since the establishment of the Association for the Study of Internal Fixation (AO) principles, rigid internal fixation has been widely accepted as the standard strategy for fracture management, particularly with the development of high-stiffness metallic plates and locking plate systems [1,2]. Although rigid fixation effectively maintains alignment and enables early mobilization, increasing evidence suggests that excessive construct stiffness may suppress interfragmentary strain, impair callus formation, and compromise secondary fracture healing. These concerns have renewed interest in fixation strategies that balance mechanical stability with biological compatibility [3,4,5].

Secondary fracture healing is a strain-dependent, callus-mediated process that relies on controlled interfragmentary motion, preserved vascularity, and coordinated cellular responses. Experimental and clinical studies have demonstrated that moderate micromotion at the fracture site promotes endochondral ossification and robust callus formation, whereas overly rigid fixation shifts load away from the bone and induces stress shielding [6,7,8,9]. In this context, the mechanical properties of fixation devices are increasingly recognized as critical determinants of the biological healing response rather than merely structural supports.

Conventional bone plates, typically manufactured from stainless steel or Titanium–6 Aluminum–4 Vanadium (Ti6Al4V) alloys, possess elastic moduli far exceeding that of cortical bone. While these materials provide excellent strength and fatigue resistance, their high stiffness often results in load-bearing rather than load-sharing fixation, limiting the transmission of physiological strain to the fracture site [10,11,12,13]. This mismatch between implant rigidity and bone biomechanics has prompted the exploration of alternative materials designed to optimize the mechanical environment for fracture healing.

**Figure 1:**
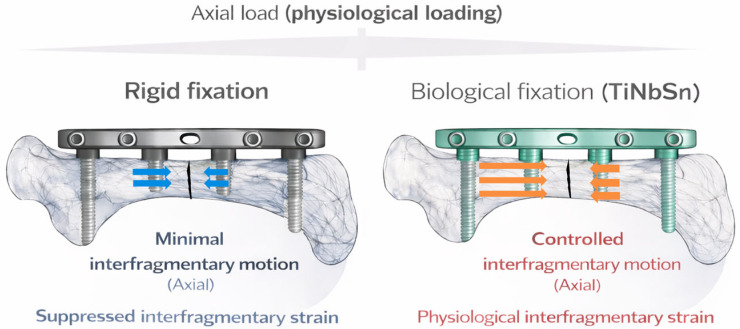
Conceptual comparison of conventional rigid fixation and low-elastic-modulus TiNbSn alloy plates. The orange arrows indicate greater interfragmentary motion at the fracture site in the TiNbSn group, whereas the blue arrows represent the corresponding motion in the control group.

Titanium–Niobium–Tin (TiNbSn) alloy is a β-type titanium alloy characterized by a Low–Young’s modulus, heat-treatment-tunable stiffness, and favorable biocompatibility [14,15,16,17]. Unlike conventional high-stiffness alloys, TiNbSn exhibits mechanical properties closer to those of bone, allowing controlled elastic deformation under physiological loading. Recent preclinical studies combining mechanical analyses and in vivo fracture models have demonstrated that TiNbSn alloy bone plates can enhance callus formation and promote more physiological fracture healing compared with conventional rigid plates [12,18]. Early material investigations demonstrated that β-type TiNbSn alloys were originally designed to achieve a low elastic modulus while maintaining mechanical strength and biocompatibility [19,20,21]. As conceptually illustrated in Figure 1, conventional high-stiffness plates primarily function as load-bearing constructs that suppress interfragmentary strain, whereas low-modulus TiNbSn plates facilitate controlled elastic deformation and physiological load sharing. However, despite growing mechanical and experimental evidence, the biological rationale underlying these outcomes has not been fully integrated into fracture fixation concepts.

Rigid plates function as load-bearing constructs with high stiffness, diverting axial load through the implant and suppressing interfragmentary strain at the fracture site. In contrast, TiNbSn alloy plates allow controlled elastic deformation and load sharing with the bone, resulting in greater axial displacement and maintenance of biologically favorable interfragmentary strain. This mechanical environment supports callus formation and secondary fracture healing.

The purpose of this review is to synthesize current preclinical evidence on TiNbSn alloy bone plates within the framework of fracture healing biology and mechanical fixation theory. By integrating strain-dependent healing mechanisms, biomechanical requirements for biological fixation, and in vivo experimental findings, this review aims to clarify why low-elastic-modulus TiNbSn alloy plates represent a paradigm shift from rigid fixation toward biologically optimized fracture stabilization. This article is intended as a narrative review that synthesizes representative experimental, computational, and preclinical studies relevant to mechanobiological fracture fixation and TiNbSn alloy plates, rather than a systematic review based on predefined database searches or screening criteria. This article is intended as a narrative review that synthesizes representative experimental, computational, and preclinical studies relevant to mechanobiological fracture fixation and TiNbSn alloy plates, rather than a systematic review based on predefined database searches or screening criteria. This focused review includes representative preclinical, computational, and translational studies selected based on their relevance to mechanobiological fracture healing and TiNbSn fixation systems.

## 2. Biological Basis of Secondary Fracture Healing

Secondary fracture healing is the predominant biological pathway following relative stability at the fracture site. It proceeds through overlapping phases of inflammation, soft callus formation, hard callus formation, and remodeling. This process is highly dependent on the local mechanical environment, particularly interfragmentary strain and vascular integrity, and represents a tightly regulated interaction between biological signaling and biomechanical stimuli [22,23,24,25].

At the cellular level, secondary fracture healing is a complex, multiscale biological process involving coordinated cellular, molecular, and tissue-level events. The periosteum serves as the primary source of skeletal progenitor cells, providing mesenchymal stem cells (MSCs) that differentiate into chondrocytes and osteoblasts to form cartilaginous and bony callus [26,27,28]. This regenerative cascade is regulated by multiple signaling pathways, including Wnt/β-catenin, TGF-β/BMP, and Indian hedgehog (Ihh)/parathyroid hormone-related protein (PTHrP), which collectively govern cell fate determination, matrix production, and ossification timing [26,29,30].

Inflammatory signaling plays a critical initiating role in fracture repair. Cytokines such as interleukin (IL)-1, IL-6, and tumor necrosis factor (TNF)-α, together with growth factors including vascular endothelial growth factor (VEGF), fibroblast growth factor (FGF), and platelet-derived growth factor (PDGF) orchestrate early cellular recruitment, angiogenesis, and tissue organization [31,32,33]. Among immune cells, macrophages have emerged as key regulators of healing progression. In particular, M2-polarized macrophages predominate during the ossification phase, and their induction via IL-4 and IL-13 has been shown to significantly enhance bone formation [34,35]. These findings highlight the intimate coupling between immune modulation and skeletal regeneration.

The mechanical environment critically determines the pathway of ossification during secondary fracture healing. Perren’s interfragmentary strain theory describes how tissue differentiation at the fracture site is governed by the magnitude of strain experienced by the healing tissue. High strain favors fibrous tissue formation, moderate strain promotes cartilage formation, and low strain allows direct bone formation [6,36]. Quantitatively, strains below approximately 5% under low hydrostatic pressure promote intramembranous ossification, whereas strains in the range of 5–15% combined with higher hydrostatic pressures stimulate endochondral ossification [37]. Experimental micromotion studies have confirmed that controlled axial displacement enhances endochondral callus maturation [38,39]. Importantly, complete elimination of strain through excessively rigid fixation may suppress the biological stimulus required for callus formation, thereby delaying or impairing healing [40].

Experimental models have consistently demonstrated that controlled micromotion enhances endochondral ossification and accelerates fracture healing [8,41]. Preservation of periosteal blood supply and avoidance of excessive stress shielding are therefore critical components of biologically favorable fixation [3]. Angiogenesis has emerged as a decisive determinant of healing success, as the establishment of a functional vascular network is essential for oxygen delivery, cell differentiation, and tissue maturation throughout the repair process [42].

Healing progresses through defined temporal phases—namely inflammation (days 0–4), soft callus formation (days 3–14), hard callus formation (days 14–28), and remodeling (weeks to months) [43,44]. However, this progression is strongly influenced by host factors. Aging, in particular, significantly delays fracture healing, with experimental studies demonstrating that elderly animals require nearly twice as long as juveniles to replace cartilaginous callus with bone [45]. Additional factors that enhance healing include cyclooxygenase-2 (COX-2) activity, which promotes MSC recruitment and angiogenesis, preservation of periosteal circulation, and flexible fixation that permits controlled interfragmentary motion [46,47]. Conversely, chronic inflammation, diabetes mellitus, cigarette smoke exposure, and advanced age are well-established contributors to delayed union and nonunion [22,48].

Collectively, these findings demonstrate that secondary fracture healing recapitulates key aspects of embryonic bone development through spatially and temporally regulated gene expression. The mechanical environment acts not merely as a passive condition but as an active regulator of cellular behavior and molecular signaling. Consequently, fixation strategies that preserve physiological strain and vascularity are essential for optimizing biological fracture repair.

## 3. Mechanical Requirements for Biological Fixation

From a biomechanical perspective, fracture fixation devices must achieve a delicate balance between stability and flexibility. The primary objective is to provide sufficient stability to prevent excessive interfragmentary motion that would lead to fibrous tissue formation or nonunion, while simultaneously allowing controlled deformation under physiological loading to stimulate biological repair. This approach, commonly referred to as biological fixation, fundamentally contrasts with traditional rigid fixation strategies that prioritize absolute stability and minimal motion at the fracture site [1].

Biological fixation is grounded in the understanding that bone is a mechanosensitive tissue whose regeneration is regulated by local strain and stress distributions. Accordingly, fixation devices should be designed not merely to immobilize the fracture, but to actively modulate the mechanical environment in a manner conducive to secondary fracture healing [49]. Three key mechanical requirements underpin this concept [50].

First, effective biological fixation requires load sharing between the implant and the bone. Ideally, the fixation construct should transmit physiological loads through both the implant and the healing bone, rather than diverting the majority of load to the implant alone [1]. Load-sharing constructs preserve mechanical stimulation of the fracture site and surrounding bone, thereby supporting callus formation and preventing stress-shielding-induced bone resorption [51]. In this review, stress shielding is discussed as a qualitative reduction in bone load transfer inferred from comparative deformation and stress patterns, rather than as a directly quantified biomechanical parameter. In contrast, load-bearing constructs concentrate stress within the implant, reducing strain transmission to the bone and potentially delaying healing.

Second, controlled elastic deformation under cyclic loading is essential. During daily activities, fracture fixation constructs are subjected to repetitive, low-magnitude loading rather than single static loads [38]. Devices capable of elastic deformation can accommodate these cyclic loads while maintaining structural integrity, allowing small but consistent interfragmentary motion [40]. These concepts parallel flexible fixation principles such as far cortical locking, which demonstrably increases callus formation by reducing construct stiffness [52,53]. Such controlled deformation has been shown to enhance endochondral ossification and promote more robust callus maturation [38]. Conversely, constructs with minimal elastic compliance may suppress these beneficial mechanical signals [50].

Third, maintenance of interfragmentary strain within a biologically favorable range is critical. As described by interfragmentary strain theory, tissue differentiation at the fracture site is dictated by the magnitude of local strain [37,49]. Excessive motion leads to fibrous tissue formation, whereas overly rigid fixation reduces strain below the threshold required to initiate callus formation. Biological fixation therefore seeks to maintain strain within an intermediate window that supports cartilage formation and subsequent endochondral bone formation throughout the healing process [37].

Fixation devices with excessive stiffness tend to function as load-bearing systems, shielding the fracture site and adjacent bone from physiological stress [51,54]. This stress shielding not only suppresses callus formation but may also induce cortical bone resorption beneath the implant, compromising long-term structural integrity. Such effects are particularly pronounced with modern locking plate constructs, which can significantly increase overall construct stiffness, especially in bridging fixation [3].

In contrast, fixation devices with lower stiffness can facilitate load sharing and strain modulation, creating a mechanical environment that more closely approximates physiological conditions [52,53]. By allowing controlled deformation while maintaining alignment and stability, these constructs support the biological processes underlying secondary fracture healing. Importantly, this approach does not imply insufficient fixation strength, but rather a shift in design philosophy—from maximal rigidity toward mechanically and biologically optimized stabilization [50].

Collectively, these considerations underscore that the mechanical success of fracture fixation should not be judged solely by initial construct stiffness or resistance to deformation. Instead, optimal fixation requires a nuanced balance in which stability, elasticity, and strain distribution are tailored to support the biological demands of fracture healing. This paradigm provides the mechanical foundation for evaluating emerging fixation materials and designs aimed at enhancing biological repair [1,50]. Similar mechanobiological concepts have also been explored in other low-modulus fixation systems, including alternative titanium alloys and polymer-based implants, which have demonstrated improved load sharing and enhanced callus formation under reduced construct stiffness [55,56,57,58,59,60].

In addition to material-based stiffness modulation using Low–Young’s modulus alloys, construct stiffness can also be reduced through structural design strategies, such as porous or lattice-based fixation systems, which have been reported to promote load sharing and favorable fracture healing environments [61,62,63]. While these structural approaches represent an important direction in mechanobiologically informed fixation design, a detailed comparison among different structural optimization strategies is beyond the scope of the present review. Instead, this article focuses on the material-driven modulation of construct stiffness using TiNbSn alloys, highlighting its specific advantages and limitations within the broader implant design landscape.

## 4. Limitations of Conventional High-Stiffness Bone Plates

Conventional bone plates manufactured from stainless steel or Ti6Al4V alloys have been the cornerstone of fracture fixation for decades. Their widespread adoption is rooted in their high strength, corrosion resistance, and excellent fatigue performance, which reliably prevent implant failure under physiological and supraphysiological loads. With elastic moduli of approximately 190–200 GPa for stainless steel and around 105–115 GPa for Ti6Al4V alloys, these materials were historically favored to ensure structural security, particularly in an era when implant breakage represented a major clinical concern [49,51,61,64,65,66].

However, the mechanical robustness of high-stiffness plates comes at the cost of a pronounced mismatch with the elastic properties of cortical bone. From a biomechanical standpoint, this mismatch fundamentally alters load transfer within the bone–implant construct. Rather than sharing load with the healing bone, excessively stiff plates preferentially bear mechanical stress, thereby reducing strain transmission across the fracture site. As a consequence, the fixation construct may satisfy mechanical stability requirements while simultaneously undermining the mechanobiological stimuli essential for secondary fracture healing [1].

This shift toward load-bearing fixation has important biological implications. Reduced interfragmentary strain suppresses callus formation and limits endochondral ossification, favoring healing pathways that depend on absolute stability [37]. While primary bone healing can occur under such conditions, it is less tolerant of biological compromise and is often suboptimal in diaphyseal fractures, comminuted patterns, or osteoporotic bone. Experimental and clinical studies have consistently associated rigid fixation with diminished callus volume, delayed union, and increased susceptibility to nonunion in these contexts [38,67].

Beyond the fracture site, stress shielding induced by high-stiffness plates affects the surrounding cortical bone. Clinical reports have described cortical thinning and delayed healing with locking plates in distal femur fractures [11,68]. By diverting physiological loading away from the cortex, rigid constructs disrupt normal bone remodeling, promoting localized bone resorption and cortical thinning beneath the plate. This phenomenon not only compromises bone quality during fixation but also creates a latent vulnerability, increasing the risk of refracture after implant removal—an issue of particular concern in elderly and osteoporotic patients [51,69].

The advent of locking plate technology has further accentuated these limitations. Locking plates form fixed-angle constructs that function as internal fixators, substantially increasing overall construct stiffness, especially in bridging fixation. Although this design has clear advantages in maintaining alignment and preventing implant failure in unstable fractures, it can inadvertently eliminate the controlled micromotion necessary for biologically favorable healing. In effect, the very features that enhance mechanical security may exacerbate biological suppression at the fracture site [1,52,53].

These observations highlight a fundamental clinical dilemma: fixation strategies optimized to maximize stiffness and stability may not necessarily optimize fracture healing biology. In many cases, surgeons must choose between mechanical security and biological efficiency, particularly when treating fractures that rely predominantly on secondary healing mechanisms. The limitations of conventional high-stiffness bone plates therefore do not reflect deficiencies in material strength or design itself, but rather a misalignment between traditional fixation priorities and the contemporary understanding of fracture healing biology [50,70].

Collectively, these considerations underscore the need to re-evaluate fracture fixation materials and constructs from a mechanobiological perspective. While high-stiffness plates remain indispensable in certain clinical scenarios, their inherent limitations in supporting biological healing have motivated the exploration of alternative fixation strategies that better balance mechanical stability with physiological strain transmission [50,71,72,73].

## 5. Material Characteristics of TiNbSn Alloy Plates

TiNbSn alloy is a β-type titanium alloy specifically designed to achieve a low elastic modulus while preserving the mechanical strength required for load-bearing orthopedic applications [15,74]. With a Young’s modulus in the range of approximately 40–60 GPa, TiNbSn alloy exhibits stiffness substantially closer to that of cortical bone than conventional fixation materials such as stainless steel or Ti6Al4V alloys. This reduced stiffness directly addresses the mechanical mismatch inherent to traditional high-stiffness bone plates [12,14,16,18,75].

Despite its low elastic modulus, TiNbSn alloy maintains sufficient yield strength, ultimate tensile strength, and fatigue resistance to withstand physiological and repetitive loading conditions encountered during fracture healing. Importantly, this balance between compliance and strength enables TiNbSn alloy plates to provide mechanical stability without excessive rigidity, thereby satisfying the fundamental requirements of biological fixation [12,14,16,18,75].

A key mechanical characteristic of TiNbSn alloy is its Low–Young’s modulus-driven elastic compliance. Under physiological loading, TiNbSn alloy plates undergo elastic deformation within the linear elastic range and return to their original configuration upon load removal, without relying on stress-induced phase transformations or shape-memory effects [12,75,76].

This elastic compliance allows TiNbSn plates to accommodate cyclic loading encountered during daily activities while maintaining structural integrity and alignment. Importantly, the deformation behavior of TiNbSn alloy reflects its reduced stiffness relative to conventional titanium alloys, rather than enhanced ductility or plastic deformation [75].

From a fixation standpoint, this low-modulus elastic response enables TiNbSn plates to transmit mechanical load to the underlying bone in a controlled manner, facilitating load sharing and strain modulation at the fracture site. As a result, TiNbSn plates can support physiological interfragmentary motion without compromising fixation stability, distinguishing them from rigid load-bearing constructs [40,75].

These mechanical characteristics allow TiNbSn alloy plates to function as load-sharing devices rather than load-bearing constructs. By elastically deforming under physiological loading, TiNbSn plates permit controlled transmission of mechanical stress to the fracture site, maintaining interfragmentary strain within a biologically favorable range. This load-sharing behavior contrasts sharply with that of high-stiffness plates, which tend to shield the bone from mechanical stimulation and suppress callus formation [40,75].

Beyond mechanical compatibility, TiNbSn alloy has demonstrated excellent biocompatibility in preclinical evaluations [17]. In vitro and in vivo studies have reported no evidence of cytotoxicity or adverse inflammatory responses under the tested experimental conditions, including specific cell types, evaluation periods, and animal models, when compared with conventional titanium-based control materials. Osteogenic cell adhesion and proliferation on TiNbSn surfaces are comparable to, or in some cases superior to, those observed with conventional titanium alloys [77,78]. These findings indicate that the alloy’s favorable mechanical behavior is not achieved at the expense of biological safety.

Crucially, the material characteristics of TiNbSn alloy should not be interpreted as representing a mechanically inferior fixation material. Rather, TiNbSn alloy plates embody a deliberate material design strategy aimed at optimizing the mechanical environment for secondary fracture healing. By combining a Low–Young’s modulus, elastic compliance within the physiological range, sufficient strength, and biocompatibility, TiNbSn alloy plates are uniquely positioned to resolve the biological–mechanical dilemma associated with conventional rigid fixation [16,18,75].

Taken together, these properties suggest that TiNbSn alloy plates represent a distinct class of fixation devices in which material behavior is intentionally aligned with the biology of fracture healing. This alignment provides the mechanistic foundation for the enhanced callus formation and more physiological healing patterns observed in preclinical fracture models.

**Figure 2:**
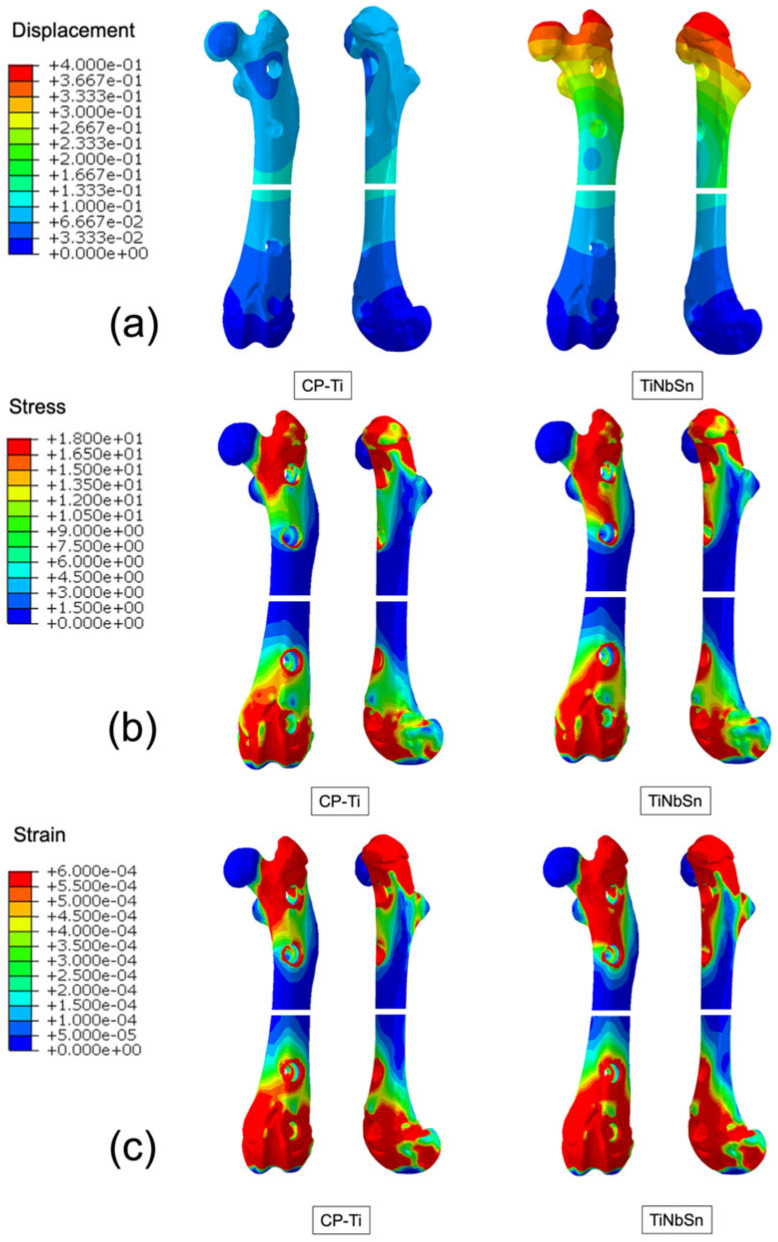
Finite element analysis of a rat femur osteotomy model stabilized with a conventional CP-Ti plate or a Low–Young’s modulus TiNbSn plate. (a) Axial displacement distribution under axial loading conditions. (b) von Mises stress distribution in the fixation construct and femur. (c) Interfragmentary strain distribution at the osteotomy site. Color scales indicate relative magnitudes for qualitative comparison. These finite element analyses illustrate trends in displacement and stress distribution associated with reduced plate stiffness and are not intended to provide a comprehensive quantitative mechanical comparison of complete fixation constructs. This figure is reproduced from our previous publication [75] with permission from the publisher. These results should be interpreted as illustrative of relative mechanical trends under simplified assumptions, rather than as direct quantitative or clinical predictions. Absolute values were not intended for quantitative comparison, and the model assumptions, including bonded contact conditions and linear elastic behavior, represent simplified conditions.

The mechanical consequences of the Low–Young’s modulus of the TiNbSn alloy are illustrated in finite element analysis models previously reported in a rat femur osteotomy model [75] (Figure 2). These finite element analyses were intended to illustrate qualitative trends in displacement and stress distribution associated with reduced plate stiffness, rather than to provide a comprehensive mechanical comparison of complete fixation constructs.

In the referenced finite element model [75], a rat femur osteotomy stabilized with either a conventional CP-Ti plate or a Low–Young’s modulus TiNbSn plate was analyzed under axial loading conditions. The analysis was performed as a linear elastic, quasi-static simulation, assuming isotropic material properties. These modeling assumptions, including bonded contact conditions and linear elastic behavior, were adopted to enable qualitative comparison of deformation and stress distribution trends, but they do not fully capture the complexity of physiological loading and bone–implant interactions.

In the present review, the term “stress shielding” is used to describe a relative reduction in load transfer from the implant to the surrounding bone, inferred from differences in stress and deformation patterns observed in the finite element analyses. In the referenced models, stress shielding was not directly quantified using dedicated metrics such as load-sharing ratios, strain energy distributions, or explicit separation of stress transfer through the plate versus bone. Rather, reduced stress concentration within the plate and increased deformation of the fixation construct were interpreted as indicative of a shift toward load sharing with the bone. The absence of quantitative stress-shielding indices represents a limitation of the current analysis and is acknowledged accordingly.

## 6. Interpretation of Preclinical In Vivo Evidence

Animal fracture models using TiNbSn alloy plates have consistently demonstrated enhanced callus formation and more physiological healing patterns compared with conventional high-stiffness plates [12,18,70]. Radiological analyses have shown increased callus volume, wider callus distribution, and earlier cortical bridging, while histological evaluations revealed prolonged preservation of cartilage callus and orderly progression of endochondral ossification rather than premature direct bone formation [12,18,70].

**Figure 3:**
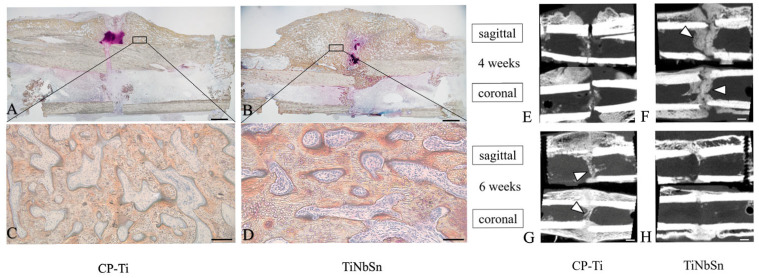
Histological and radiological assessment of fracture healing following fixation with low-elastic-modulus TiNbSn plates in rabbit tibial osteotomy models. (**A**,**B**) Low-magnification non-decalcified histological sections at 4 weeks demonstrate increased periosteal and intramedullary callus formation in the TiNbSn group compared with conventional rigid fixation. (**C**,**D**) High-magnification images at 4 weeks reveal active endochondral ossification with abundant cartilage, woven bone, and osteoid in the TiNbSn group. (**E**,**F**) CT images at 4 weeks show extensive periosteal callus and intramedullary new bone (arrowheads) with TiNbSn fixation. (**G**,**H**) CT at 6 weeks demonstrates further consolidation and progression of intramedullary ossification in the TiNbSn group. Together, these findings indicate enhanced and more physiologic secondary fracture healing with TiNbSn plates. Triangles indicate bone formation within the bone marrow. This figure is reproduced from our previous publication [12] with permission from the publisher.

As shown in Figure 3, these mechanical predictions are reflected in the rabbit tibial fracture model, where TiNbSn fixation resulted in greater periosteal and intramedullary callus formation compared with rigid plates.

From a mechanobiological perspective, these findings can be interpreted as the consequence of a more favorable local strain environment at the fracture site [50,70]. The reduced elastic modulus of TiNbSn plates allows controlled elastic deformation under physiological loading, resulting in moderated interfragmentary strain that remains within the optimal window for secondary bone healing [50,70]. This mechanical milieu supports mesenchymal cell differentiation toward chondrogenic and osteogenic lineages, while preserving vascular ingrowth essential for callus maturation [23,50].

Importantly, the observed enhancement of fracture healing does not appear to reflect a direct osteoinductive effect of the TiNbSn alloy itself [23,70]. Rather, TiNbSn plates function as mechanical regulators, mitigating stress shielding and preventing excessive rigidity that can suppress callus formation and vascular perfusion [23,70,75]. By maintaining load sharing between the implant and the healing bone, these plates enable intrinsic biological repair mechanisms to proceed in a spatially and temporally coordinated manner [23,50,70,75]. Representative preclinical studies supporting the mechanobiological effects of Low–Young’s modulus TiNbSn fixation systems are summarized in Table 1.

Taken together, preclinical in vivo evidence suggests that TiNbSn alloy plates promote fracture healing not by accelerating bone formation itself, but by establishing a mechanically permissive environment that closely approximates physiological loading conditions [12,18,23,50,70,75,76]. This concept aligns with the principles of biological fixation, in which controlled micromotion and optimized strain distribution are used to facilitate robust, reproducible secondary bone healing [6,23,50]. It should be noted that comparability across preclinical studies is influenced by heterogeneity in animal species, implant geometry, screw configuration, and loading conditions.

Despite these potential advantages, low-modulus fixation systems may also present certain limitations. Reduced construct stiffness may compromise initial mechanical stability in highly unstable fracture patterns or in cases with poor bone quality. In addition, excessive interfragmentary motion under certain loading conditions may impair fracture healing rather than promote it. Therefore, careful consideration of fracture type, fixation strategy, and mechanical environment is essential when applying low-modulus implants in clinical practice.

## 7. Potential Clinical Implications

Although current evidence for TiNbSn alloy plates is primarily derived from preclinical studies, the biological fixation concept underlying Low–Young’s modulus TiNbSn alloys is supported by emerging clinical data from other orthopedic applications [79,80,81,82,83]. In particular, TiNbSn alloy femoral stems have been clinically applied as the TNS stem, with mid-term follow-up results of up to seven years demonstrating sustained implant stability and a marked reduction in stress shielding [79,80,81].

It should be emphasized that fracture fixation plates and femoral stems differ substantially in implantation site, loading mode, and bone–implant interface. Femoral stems are primarily subjected to long-term axial and bending loads within the intramedullary environment, whereas fracture plates experience more complex and variable loading conditions at the cortical surface. Therefore, clinical outcomes of TiNbSn femoral stems should be interpreted as indirect and supportive evidence for the mechanobiological advantages of Low–Young’s modulus titanium alloys, rather than as direct validation of plate fixation performance.

These clinical findings suggest that TiNbSn alloys can maintain physiological load transfer and bone remodeling over time [81], providing indirect but important clinical support for their application in fracture fixation. In the context of plate osteosynthesis, such properties may be especially advantageous in diaphyseal fractures, osteoporotic bone, and fractures at high risk for delayed union or nonunion, where excessive construct stiffness is known to compromise biological healing.

Furthermore, reduced stress shielding associated with low-elastic-modulus implants may contribute to the preservation of bone stock and potentially decrease the risk of refracture after plate removal [75]. The integration of TiNbSn alloys with modern plate designs, including locking mechanisms, therefore represents a promising strategy for next-generation fracture fixation systems that aim to balance mechanical stability with biological healing.

At present, although TiNbSn alloy femoral stems have been introduced into clinical practice, TiNbSn plates for fracture fixation are not yet widely available in routine clinical use and remain primarily at the preclinical stage. Further regulatory approval and clinical validation will be required before their broader clinical adoption.

## 8. Limitations and Future Perspectives

The primary limitation of the current body of evidence is the paucity of long-term clinical data directly evaluating TiNbSn alloy plates in fracture fixation. Most available insights are derived from animal models, which, although informative, cannot fully replicate the complex biomechanical environment, loading patterns, and biological variability present in human fracture healing. Differences in bone quality, fracture configuration, and postoperative rehabilitation between experimental models and clinical practice must therefore be carefully considered when extrapolating preclinical findings [84,85].

Another important limitation is the heterogeneity of experimental designs, including variations in plate geometry, fixation constructs, and loading conditions, which complicates direct comparison across studies. Furthermore, the optimal balance between construct stiffness and controlled micromotion remains incompletely defined and is likely to vary depending on fracture location, bone quality, and patient-specific factors [85,86].

It should also be noted that a substantial proportion of the referenced studies originate from our research group, which may introduce a degree of selection bias despite efforts to include representative literature.

Future research should prioritize well-designed prospective clinical studies to validate whether the mechanobiological advantages observed in preclinical models translate into improved clinical outcomes, such as reduced rates of delayed union, nonunion, and refracture. In parallel, optimization of plate geometry and fixation strategies, including screw configuration and locking mechanisms, will be essential to fully exploit the low elastic modulus properties of TiNbSn alloys. In addition, computational modeling and finite element analysis may play a critical role in characterizing local strain distributions and guiding the development of strain-controlled fixation systems tailored to specific clinical scenarios.

Collectively, these efforts may enable the transition from empirically designed fixation constructs toward mechanistically informed, patient-adapted fracture fixation, in which implant material properties and structural design are intentionally integrated to support biological healing.

## 9. Conclusions

TiNbSn alloy bone plates represent a material-based approach to biologically informed fracture fixation grounded in mechanobiological principles. The present review synthesizes current evidence to clarify their potential role and limitations.

First, established mechanobiological concepts indicate that controlled interfragmentary strain, load sharing, and preservation of vascularity are critical determinants of secondary fracture healing. Excessive construct stiffness associated with conventional high-stiffness plates may suppress these biological processes.

Second, preclinical evidence from finite element analyses and animal fracture models consistently suggests that Low–Young’s modulus TiNbSn plates allow greater elastic compliance, promote load sharing, and maintain interfragmentary strain within a biologically favorable range, resulting in enhanced callus formation and more physiological healing patterns.

Third, available clinical experience with TiNbSn femoral stems provides indirect and supportive evidence for the long-term benefits of low-elastic-modulus titanium alloys in mitigating stress shielding; however, these findings should be interpreted only as indirect supportive evidence, since stem implantation and fracture plate fixation involve substantially different mechanical and biological contexts.

Finally, while the current body of evidence provides preliminary support for the mechanobiological rationale of TiNbSn alloy plates, further well-designed clinical studies are required to define their optimal indications and to translate these findings into clinical practice.

## Figures and Tables

**Table 1:** Summary of representative preclinical studies evaluating Low–Young’s modulus TiNbSn alloy fixation systems.

Study	Model	Fixation System	Evaluated Mechanical Aspects	Observed Biological Findings	Relevance to Low-Modulus Fixation
Fujisawa et al., 2018 [14]	Mouse tibia fracture	TiNbSn intramedullary nail	Low-modulus elastic nail	Increased callus formation	Biological compatibility of elastic fixation
Mori et al., 2021 [15]	Mouse tibia fracture	TiNbSn intramedullary nail	Elastic intramedullary fixation	Enhanced endochondral ossification	Healing patterns observed under low-modulus fixation
Kogure et al., 2019 [16]	Rabbit tibial osteotomy	TiNbSn elastic nail	Reduced construct stiffness	Increased periosteal callus	Tolerance of controlled elastic fixation
Ito et al., 2022 [18]	Rabbit tibial osteotomy	TiNbSn plate	Favorable stress distribution	Greater callus volume	Association between plate stiffness and callus formation
Koguchi et al., 2023 [12]	Rabbit tibia osteotomy	TiNbSn locking plate	Increased axial displacement	Accelerated osteosynthesis	Consistency with load-sharing fixation concepts
Koyama et al., 2025 [75]	Rat femur osteotomy	TiNbSn plate (FEA + in vivo)	Maintained strain within optimal range	Enhanced callus formation	Qualitative support for mechanobiological rationale

## Data Availability

No new data were created or analyzed in this study. Data sharing is not applicable to this article.

## Abbreviations

The following abbreviations are used in this manuscript:

AO	Association for the Study of Internal Fixation
Ti6Al4V	Titanium–6 Aluminum–4 Vanadium
TiNbSn	Titanium–Niobium–Tin
MSCs	Mesenchymal stem cells
Wnt	Wingless/Integrated pathway
TGF-β	Transforming growth factor beta
BMP	Bone morphogenetic protein
Ihh	Indian hedgehog
PTHrP	Parathyroid hormone-related protein
IL	Interleukin
TNF-α	Tumor necrosis factor alpha
VEGF	Vascular endothelial growth factor
FGF	Fibroblast growth factor
PDGF	Platelet-derived growth factor
COX-2	Cyclooxygenase-2
CP-Ti	Commercially pure titanium
FEM	Finite element analysis / Finite element method
CT	Computed tomography
